# Regeneration Patterns of European Oak Species (*Quercus petraea* (Matt.) Liebl., *Quercus robur* L.) in Dependence of Environment and Neighborhood

**DOI:** 10.1371/journal.pone.0134935

**Published:** 2015-08-12

**Authors:** Peter Annighöfer, Philip Beckschäfer, Torsten Vor, Christian Ammer

**Affiliations:** 1 Department Silviculture & Forest Ecology of the Temperate Zones, University of Göttingen, Büsgenweg 1, 37077 Göttingen, Germany; 2 Chair of Forest Inventory & Remote Sensing, University of Göttingen, Büsgenweg 5, 37077 Göttingen, Germany; Chinese Academy of Forestry, CHINA

## Abstract

*Quercus robur* L. (pedunculate oak) and *Quercus petraea* (Matt.) Liebl. (sessile oak) are two European oak species of great economic and ecological importance. Even though both oaks have wide ecological amplitudes of suitable growing conditions, forests dominated by oaks often fail to regenerate naturally. The regeneration performance of both oak species is assumed to be subject to a variety of variables that interact with one another in complex ways. The novel approach of this research was to study the effect of many ecological variables on the regeneration performance of both oak species together and identify key variables and interactions for different development stages of the oak regeneration on a large scale in the field. For this purpose, overstory and regeneration inventories were conducted in oak dominated forests throughout southern Germany and paired with data on browsing, soil, and light availability. The study was able to verify the assumption that the occurrence of oak regeneration depends on a set of variables and their interactions. Specifically, combinations of site and stand specific variables such as light availability, soil pH and iron content on the one hand, and basal area and species composition of the overstory on the other hand. Also browsing pressure was related to oak abundance. The results also show that the importance of variables and their combinations differs among the development stages of the regeneration. Light availability becomes more important during later development stages, whereas the number of oaks in the overstory is important during early development stages. We conclude that successful natural oak regeneration is more likely to be achieved on sites with lower fertility and requires constantly controlling overstory density. Initially sufficient mature oaks in the overstory should be ensured. In later stages, overstory density should be reduced continuously to meet the increasing light demand of oak seedlings and saplings.

## Introduction

The two native mid-successional European oak species *Quercus robur* L. and *Quercus petraea* (Matt.) Liebl., commonly referred to as pedunculate oak and sessile oak, are among the most frequent tree species in Central Europe [1,2]. Both species are not only of great ecological interest–known for their importance as habitat and food source for a great variety of insects, mammals, birds, fungi, lichens, and moss species [3–8]–but also of considerable economic value for forest enterprises and the wood processing industry.

In Germany, oak species can be found on around 10% of the forested area, making oaks the second most important deciduous tree species [9] after European beech (*Fagus sylvatica* L.). Various German forest enterprises have the objective to increase the proportion of deciduous species on managed forest land [10] to increase the resilience of the forests. Oaks play a key role in this process, as their amplitude of suitable growing conditions is rather wide [11,12]. This is especially advantageous with regard to changing climatic and environmental conditions [13].

Both oaks can be found on soils with different cation exchange capacity and nutrient supply, from siliceous substrates to limestone soils [1,14] but show best growth on sites with moderate growing conditions and nutrient supply, where they are, however, usually outcompeted by European beech, without human intervention. Under natural conditions, oaks are therefore often found on sites with somewhat more extreme water conditions. In the case of *Q*. *robur* these can then be moist or wet and the species even survives flooding up to a certain extent [15–17]. The soils on these sites are often base and nutrient rich loam or clay soils. *Q*. *petraea* is then often rather found on well-drained shallow, stony and rocky, dry soils because the species is more sensitive to high groundwater levels and stagnating wetness [14,18] but more tolerant to drought [19]. Climatically, *Q*. *robur* has a wider amplitude than *Q*. *petraea*. While both species are well adapted to an Atlantic, sub-Mediterranean climate with mild winters, only *Q*. *robur* grows well under oceanic and continental climate conditions. Therefore, *Q*. *robur* is found further to the east, north, and south than *Q*. *petraea*. In Bavaria, *Q*. *robur* grows up to an elevation of 970 m a.s.l., where *Q*. *petraea* only reaches 715 m a.s.l. [1,14]. However, apart from extreme site conditions, both oak species are often found side by side (sympatric) in many regions of Central Europe (e.g. [14,20]); also in the study region both oaks grow sympatric and were therefore studied together here. Common to both species is that they are rather light demanding compared to other European tree species, and are therefore considered to be only moderate shade tolerant [21].

One problem in cultivating oaks in Central Europe and North America is that oak forests frequently fail to regenerate naturally [22–26]. In mast years, high acorn production often results in high seedling densities (e.g. [27]) but of these only few [28], if any survive. Therefore, artificial regeneration approaches, like planting and sowing, still are commonly applied [21,29]. Modern silvicultural planning, however, favors natural regeneration of species, as it is associated with lower costs and a vital and site adapted regeneration.

The regeneration of oak has been intensively studied [30] around the world, nevertheless, reasons for the varying success of natural oak regeneration are still not well understood. The theoretical background for this assumption is that the regeneration performance of oak seems to be subject to a variety of variables: before germination, acorn predation seems to be of large relevance for failing oak regeneration [31,32], later competition, insect pests (e.g. *Tortrix viridana* L.), fungi (e.g. *Microsphaera alphitoides* Griffon and Maubl.), water supply, light availability, and browsing, are among the most important variables [33,34]. Natural and silvicultural disturbances, are considered to promote the natural regeneration of oak [35,36].

So far, variables affecting the regeneration of both oak species were mainly studied separately at seedling stage and under controlled conditions [30]. However, in the forest many different variables interact with one another in complex ways. Specifically, knowledge of the potential interaction between site fertility and shade tolerance might offer new options for the silvicultural management of oak, e.g., it may not be necessary to open the canopy cover as fast on rich sites. Therefore, this research project combined inventory data with data on environmental and stand structural characteristics, aiming at identifying key variables influencing the performance of different development stages of natural oak regeneration in situ. To our knowledge, such a large-scale study considering a wide range of potentially influential factors has not been conducted for oak in Central Europe. The research questions leading our work were:

Can general environmental and structural forest characteristics be identified that are favorable for the regeneration of both oaks on a large scale?

Do the forest conditions promoting oak seedlings and saplings differ among the different development stages of the regeneration?

## Methods

### Study area

Three study areas were established in the Bavarian state forest enterprises Arnstein (49°58 N, 09°58 E), Ebrach (49°50 N, 10°29 E), and Kaisheim (48°46 N, 10°47 E) in southern Germany. The permission to work in all three forest enterprises the area was granted by the Bavarian State Forest Enterprise.

The altitude of the study areas ranges from around 300 m– 500 m a.s.l. The climate of the region is considered to be humid temperate. The study areas have an average annual temperature of about 9°C, with close to 14.5°C during the growing season (April–September). The annual precipitation ranges from 617 mm (Arnstein) over 709 mm (Ebrach) to 770 mm (Kaisheim) of which 330 mm– 400 mm falls during the growing season.

The main bedrock in Arnstein is lower Keuper (mainly dolostone, shales or claystones, and evaporites), partly Gipskeuper (gypsum Keuper) or lower Muschelkalk (shellbearing limestone; mainly limestone, calcareous marls, and clayey marls). In Ebrach it is Sandsteinkeuper (sandstone Keuper) and Gipskeuper, and in Kaisheim it is Bunte Trümmermassen (mainly limestone and clay minerals). Depending on the main bedrock, soils with differing nutrient supply and water holding capacities have evolved.

Species composition, and horizontal and vertical stand structure varies slightly in the study areas, due to site conditions and management history, but all chosen study sites were located in forests which were comparable with one another. All stands were classified as oak stands (≥ 70% oak basal area in the overstory on total stand level, according to forest management plans). The stands in Arnstein and Ebrach had an average age of 160 years (min = 124, max = 190), whereas those in Kaisheim were 120 years old (min = 94, max = 165) on average. The overstory had a mean basal area (G) of 22 m^2^ ha^-1^ in Arnstein, 28 m^2^ ha^-1^ in Ebrach, and 30 m^2^ ha^-1^ in Kaisheim. The trees had a mean diameter at breast height (dbh) of 32 cm (23 cm– 40 cm), 33 cm (22 cm– 43 cm), 33 cm (27 cm– 41 cm) and a dominant height of 27.6 m, 29.8 m, and 30.5 m in Arnstein, Ebrach, and Kaisheim, respectively. All stands have been managed to promote natural oak regeneration by canopy tree removal. The basal area reduction in Arnstein was stronger than in both other study areas.

### Sampling design

The three study areas (Arnstein, Ebrach, Kaisheim) cover a North–South gradient of oak distribution in Bavaria. To select stands also representing a gradient of nutrient and water supply level, sites were qualitatively assigned to four site conditions (“NW”, “Nw”, “nW”, “nw”) in advance, according to site classification maps or morphological soil classification maps. Assigned site condition consisted of a combination of nutrient supply level (“N” or “n”) and water supply level (“W” or “w”). Both characteristics were classified as being either “favorable” (upper case letters) or “unfavorable” (lower case letters), relative to average values of the region.

Within each study area and per site condition, the oldest and–among these–the four largest oak stands were selected, which were in the silvicultural stage to be naturally regenerated. This resulted in a total of 16 selected forest stands per study area. In each stand one transect plot was established for data collection (48 plots in total). Each transect plot consisted of 10 subplots and was oriented in the direction of the widest stand extent to cover the variability of occurring forest conditions as good as possible. The subplots had a spacing of 50 m to one another. Subplots straddling forest or skidding roads were placed next to the road to avoid effects related to the roads. Each subplot consisted of three concentric circular sample areas: P1 = 10 m^2^ (radius ~ 1.78 m), P2 = 250 m^2^ (radius ~ 8.92 m), and P3 = 500 m^2^ (radius ~ 12.62 m).

### Data collection

Regeneration and trees with dbh < 7 cm were recorded in P1, trees with 7 cm ≤ dbh < 20 cm in P2, and trees with dbh ≥ 20 cm in P3. Since there was nearly no ground vegetation competing with the tree regeneration in the understory, only woody species were measured and identified by species. In P1 the browsing condition of all species was recorded and the oak species were assigned to four development stages according to their height: 0 < Oak1 ≤ 20 cm, 20 cm < Oak2 ≤ 50 cm, 50 cm < Oak3 ≤ 130 cm, and 130 cm < Oak4 < dbh = 7 cm. Individuals assigned to the first development stage (Oak1) were considered as seedlings. Individuals of the second development stage (Oak2) were considered as young saplings that have reached browsing height. The third development stage (Oak3) comprised saplings still in browsing range but by far established individuals. Finally, individuals of the fourth development stage (Oak4) were considered to be older saplings out of browsing range and fully established.

Browsing condition only distinguished between browsed (browsed on side shoots, browsed on main shoot, or both) and unbrowsed trees. From this, the percentage of browsed trees (br) was calculated. All trees in P1 were considered part of the regeneration. In P2 dbh was measured aside of identifying the species. In P3 additionally the height of three most dominant trees (h3) was measured. All trees recorded in P2 and P3 were considered trees of the overstory.

To specify the four qualitative site conditions, soil samples were taken on the first and fifth subplot of each transect (96 soil sampling locations) to determine the cation exchange capacity (CEC), soil pH, and element content of the soil. Field capacity (fc) was determined by conducting centered measurements on each transect. Mineral soil was sampled in three depths, depth I = 0 cm– 10 cm, depth II = 11 cm– 20 cm, and depth III = 21 cm– 40 cm using a “Pürckhauer” driller. To determine the CEC, soil samples were air-dried, sieved (< 2 mm), and percolated with unimolar NH_4_Cl [37]. Soil pH was measured, using a pH-meter (inoLab), and element contents in mmol(+)∙kg^-1^ (dry matter) of aluminium (Al^3+^), calcium (Ca^2+^), iron (Fe^3+^), potassium (K^+^), magnesium (Mg^2+^), and sodium (Na^+^) were determined by flame photometry, using a ICP-OES-spectrometer (Spectro). Soil pH and pH after percolation allowed calculating the proton (H^+^) concentration. Field capacity (fc) was determined up to a depth of 100 cm according to the methods of German site mapping system [38]. Thereby, fc is calculated in the field by combining data on precipitation, exposition, soil depth and width, texture, compaction, etc. of the different soil horizons.

Light conditions below canopy were measured in the center of each subplot (n = 480) using a Solariscope (Behling SOL300) which provides values for direct site factor (dsf), indirect site factor (isf), total site factor (tsf), and openness (opn) by analyzing hemispherical photographs. Solariscope readings were conducted in 1.5 m above the forest floor. Data was collected from 2011 – 2013.

### Data analyses

The plot level data was analyzed separately for the total oak regeneration (OakT) and for the oak regeneration in each of the four development stages (Oak1, Oak2, Oak3, Oak4).

To identify highly correlated variables (r ≥ 0.8), we calculated Spearman's rank correlation coefficient or Pearson’s r for linear correlation. Highly correlated variables were pH and H^+^ (r = -0.87, p < 0.0001), CEC and Ca^+^ (r = 0.96, p < 0.0001), and isf with dsf and tsf (r = 0.99, p < 0.0001; r = 0.96, p < 0.0001). Hence, H^+^, Ca^+^, tsf, and dsf were excluded from the set of variables used in the analyses (Table 1).

**Table 1:** Variables used to explain the performance of oak regeneration. Species in the overstory and regeneration are distinguished by capital and lower case letters, respectively.

Explanatory variable	Abbreviation	Unit	Range (min—max)	
***Neighborhood*:**				
Ash regeneration	ash	n m^-2^	0	12.1
Beech regeneration	bch	n m^-2^	0	3.6
Hornbeam regeneration	hbm	n m^-2^	0	12.9
Maple regeneration	map	n m^-2^	0	7
Other coniferous species regeneration	ocs	n m^-2^	0	0.1
Other deciduous species regeneration	ods	n m^-2^	0	1.9
Oak trees in the overstory	OAK	n ha^-1^	18	238
Ash in the overstory	ASH	n ha^-1^	0	84
Beech in the overstory	BCH	n ha^-1^	0	472
Hornbeam in the overstory	HBM	n ha^-1^	0	384
Maple in the overstory	MAP	n ha^-1^	0	104
Other coniferous species in the overstory	OCS	n ha^-1^	0	98
Other deciduous species in the overstory	ODS	n ha^-1^	0	296
***Basal area*:**				
Total	G	m^2^ ha^-1^	14.407	40.7
Oak trees in the overstory	G_OAK	m^2^ ha^-1^	2.269	29.2
Other species in the overstory	G_OSP	m^2^ ha^-1^	2.064	29.8
***Stand parameters*:**				
Stand age	age	years	94	190
Percentage of browsed trees	br	%	0.3	54.5
Shannon diversity	H_div	numeric	0.61	1.58
***Light conditions*:**				
indirect site factor	isf	%	4	36
openness (cone angle 15°)	opn	%	2	51
***Soil conditions*:**				
pH-value	pH	numeric	3.79	5.82
Cation exchange capacity	CEC	cmol kg^-1^	39.89	305.96
Field capacity	fc	mm m^-2^	62	212
Sodium	Na	mmol kg^-1^	0.15	1.02
Potassium	K	mmol kg^-1^	0.72	4.7
Magnesium	Mg	mmol kg^-1^	0.83	92.67
Iron	Fe	mmol kg^-1^	0	5.18
Manganese	Mn	mmol kg^-1^	0.09	3.15
Aluminium	Al	mmol kg^-1^	0.66	64.09

Group comparisons between the study areas of the number of oaks on the subplots were conducted by applying the Kruskal-Wallis rank sum test. If tested significant, pairwise comparisons between groups were conducted using the Wilcoxon rank sum tests with Bonferroni adjustment. Non-parametric tests were applied because the data was not normally distributed (visual verification, Shapiro-Wilk test) and variance was not homogenous (Levene’s test).

A direct gradient analysis, Canonical Correspondence Analysis (CCA), was conducted to reveal general vegetation patterns in dependence of all collected variables. A species by plot matrix was compiled and analyzed. It was assumed that the important gradients were known and measured, also species response to the variables was assumed to be non-monotonic or unimodal. Significance of the constraints was tested using permutation tests. Significance was tested for all terms separately and for the first 16 axes. CCA was applied using the R package “vegan”, version 2.0–9 [39].

To reduce the number of explanatory variables used in the regression analysis, a Boruta analysis was conducted beforehand, to identify variables relevant for oak regeneration (R package “Boruta”, version 2.1.0, [40]). Boruta is a wrapper algorithm built around the random forest algorithm [41] implemented in the R package randomForest [42]. Random forest assesses the importance of explanatory variables through an internal cross validation and Boruta extends this approach by comparing each variable’s importance with that of a shadow variable, created by a permutation of the variable’s values [40]. This results in a classification of variables into relevant, tentative, and irrelevant variables. The classification was based on 10000 random forest runs. Variables were only considered as important if they had importance values above the highest shadow values and were then ranked depending on the fraction of random forest runs in which the variable was more important than the most important shadow variable. Separate Boruta analyses were conducted for the four development stages of oak regeneration Oak1, Oak2, Oak3, Oak4, and the total oak regeneration OakT. A maximum of the five most important variables identified by the Boruta analysis was used in a linear regression analysis. To apply the multiple regression, response variables were logarithmically transformed using x’ = log (x + 1) to normalize the response variables and the Shapiro-Wilk test was performed to test normality. All combinations of possible full models (containing all important predictor variables in varying order and their interactions) were reduced using the function “selMod” from the R package “pgirmess”, version 1.5.9 [43]. Models with deltAICc < 3 were selected and compared using the corrected AIC for finite sample sizes (AICc). Best models were further simplified to the minimal adequate model applying stepwise deletion (backward selection) of non-significant variables and interactions [44]. Non-significant variables were removed according to their p-values and models were compared using ANOVA with an F-test. If variable deletion caused a significant deviance increase, model simplification was reversed and the more complex model was assumed to be valid.

All statistical analyses, fittings, graphs, and validation tests were processed using the free software environment R [45].

## Results

### Inventory of forest structure, species, and regeneration

A total of 36383 individual trees was recorded of which 30014 belonged to the regeneration and 6369 to the overstory.

Regeneration was found on 466 subplots (97%), oak regeneration was found on a total of 314 subplots (65%). Most subplots with regeneration were found on sites preliminary classified as “NW” (218 subplots), followed by “Nw” (87), “nW” (84), and “nw” (77). The oak regeneration mainly consisted of individuals from development stage Oak1 (4718 corresponding to 61%), followed by development stage Oak2 (2191 or 28%), development stage Oak3 (726 or 9%), and development stage Oak4 (83 or 1%).

Over all study regions, an average of 1.6 oaks m^-2^ was found per subplot, with averages of 2.1 oaks m^-2^ in Arnstein, 2.2 oaks m^-2^ in Ebrach, and 0.5 oaks m^-2^ in Kaisheim. The mean number of oak regeneration per transect differed significantly between Arnstein and Kaisheim (p < 0.05) and Ebrach and Kaisheim (p < 0.05). No significant differences were found between Arnstein and Ebrach (p = 0.87).

### Relations between explanatory variables and regeneration (CCA)

**Fig 1:**
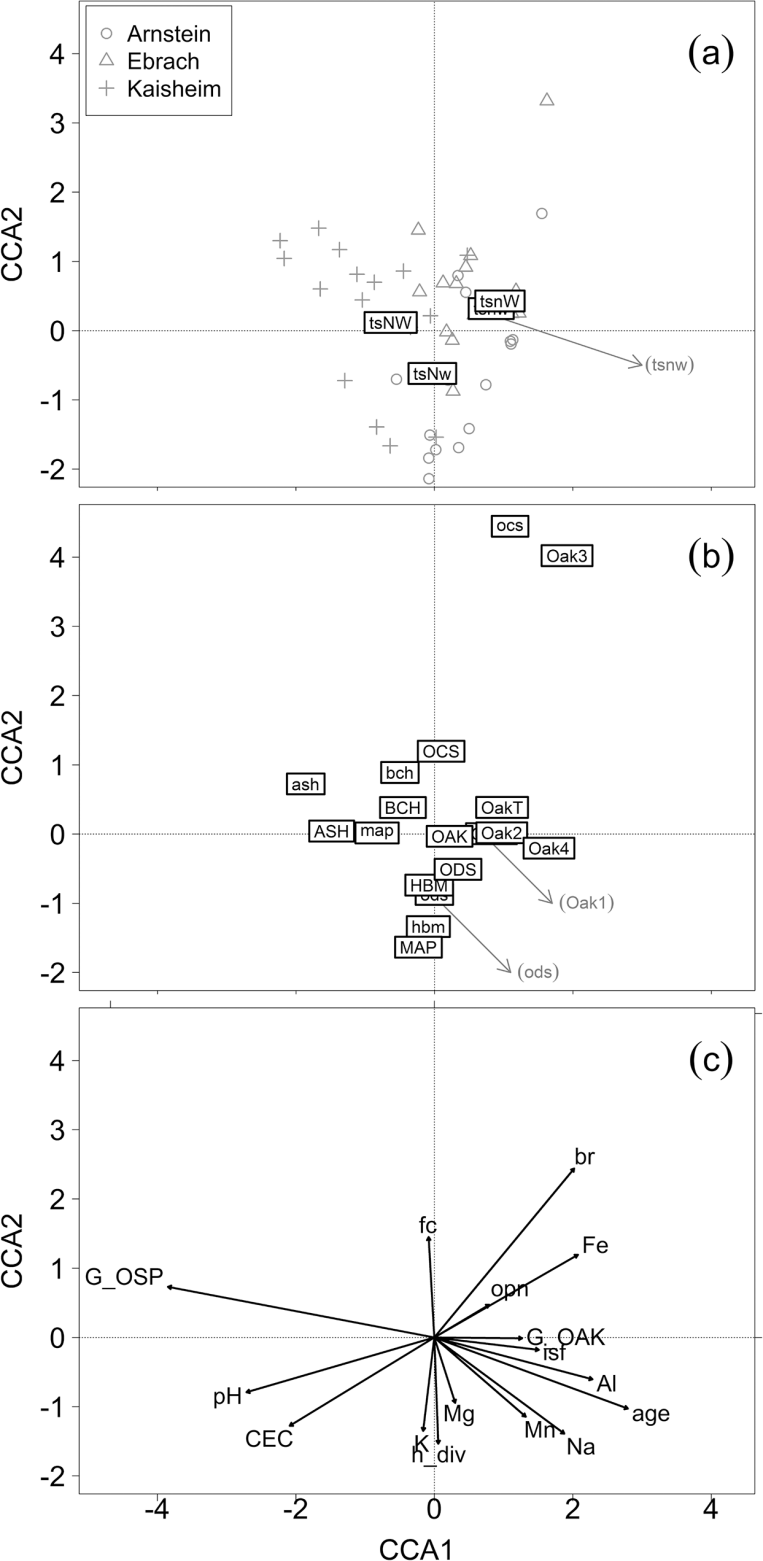
Canonical Correspondence Analysis (CCA) for the 48 plots (a), the species (b) and the environmental variables fit to the ordination (c). The four development stages of oak are labeled as Oak1, Oak2, Oak3, and Oak4, total oak regeneration is labeled as OakT, the other labels follow the abbreviations given in Table 1.

The CCA ordination in Fig 1A shows the plot and study area ordination together with nutrient and water supply levels. The ordination placed the oak regeneration on the right side of the CCA (Fig 1B). The development stage Oak3 stood alone, the other development stages were grouped together close to the first CCA axis and stood opposite to the ash and maple regeneration referring to the first axis. The other deciduous and coniferous species, and also hornbeam, seemed to be more strongly associated with the second axis. The environmental variables (Fig 1C) showed that an increase of oak regeneration can be expected with increasing light availability (isf), stand age, and basal area of oak (G_OAK). The soil conditions showed that more oak regeneration could be expected were Al and Fe contents were higher. Also the sites classified as unfavorable with respect to nutrient supply (nW, nw) were located in the direction of increasing oak regeneration. Oak regeneration in development stage Oak3 seemed to be related to browsing (br). Ash and maple regeneration were associated with more favorable site conditions (NW) and found in the direction of increasing CEC soil content and increasing pH-value.

The permutation test of all constraints simultaneously resulted in p <0.05. Testing the variables separately showed that G_OAK (p < 0.05), G_OSP (p < 0.05), and br (p < 0.05) were significant. The first five axes of the CCA had significant loadings. The Eigenvalue of the first two constrained axes were 0.449 and 0.246, respectively.

### Identification of relevant explanatory variables for the regeneration

The Boruta analysis confirmed and specified the results of the CCA analysis. A detailed inspection of the results from the Boruta analysis showed that there were differences among the variable importance for the total data and the different development stages of oak (Table 2).

**Table 2:** Variables identified as important by the Boruta analysis. Variables presented for predicting the abundance of total oak regeneration (OakT) and the regeneration in each development stage (Oak1 –Oak4), presented with their fraction of random forest runs (Norm Hits) in which they were more important than the most important shadow value.

Regeneration development stage	Identified variables	Norm Hits
OakT	br	0.95
	G_OSP	0.93
	pH	0.73
	Fe	0.71
Oak1	Fe	0.98
	G_OAK	0.81
	G_OSP	0.74
Oak2	isf	0.97
	br	0.96
	bch	0.92
	BCH	0.88
	opn	0.71
Oak3	br	1.00
	pH	0.96
	G	0.91
	ocs	0.74
Oak4	G	1.00
	G_OSP	0.94
	opn	0.87
	isf	0.75

Focusing on the overall regeneration performance of oak showed that the browsing percentage (br) and the basal area of all other species (G_OSP) were closely related to the abundance of oak regeneration. In addition, the soil pH and iron content (Fe) were identified as important variables.

Among the four development stages the importance of the variables shifted. For oaks of the first development stage (Oak1), Fe was identified as most important, followed by the measures for basal area (G_OAK, G_OSP, G). For Oak2 mainly light (isf, opn), browsing percentage (br), and competition (bch, BCH) were important. Browsing percentage was also important for Oak3, combined with soil pH and basal area (G). Eventually, for Oak4 only basal area (G, G_OSP) and light (opn, isf) were identified as important (Table 2).

### Explanatory strength of relevant variables (regression models)

The regression models reached a R^2^ value of 0.51 on average (Table 3). The deletion of non-significant variables led to models with a minimum of two (Oak1) or three (OakT) explanatory variables. For Oak2 isf was not significant but model simplification lead to significant slopes (p < 0.001) for model comparisons with ANOVA (“F-test”) justifying retaining the more complicated model, with isf as non-significant variable. The p-values of the variables of Oak3 indicated significant effects for browsing (br) and the interaction of basal area and browsing (G:br), but not for basal area alone (G). Retaining the non-significant variable for Oak3 led to the highest R^2^ of 0.832 among all development stages. The same was true for the model of Oak4, where the basal area alone was not significant, but was significant in interaction with other explanatory variables, next to the isf. Table 3 shows that different variables were of importance for the different development stages of oak regeneration, as also suggested by the Boruta analysis.

**Table 3:** Model coefficients with estimates and p-values of the regression models. Models for logarithmically transformed values of total oak regeneration (OakT) and regeneration in each development stages (Oak1 –Oak4), presented together with the adjusted R^2^ of the models.

Model	Coefficient	Estimate	p-value	Adj. R^2^
OakT	Intercept	2.5346	<0.0001	0.47
	br	0.0296	<0.05	
	G_OSP	-0.0825	<0.001	
	Fe	0.2446	<0.05	
Oak1	Intercept	2.1548	<0.0001	0.367
	Fe	0.2889	<0.001	
	G_OSP	-0.0664	<0.05	
Oak2	Intercept	0.6609	<0.05	0.464
	BCH	-0.0032	<0.05	
	br	0.0346	<0.001	
	isf	0.0329	0.064	
Oak3	Intercept	-0.1908	0.483	0.832
	G	0.0041	0.666	
	br	0.1326	<0.0001	
	G:br	-0.004	<0.0001	
Oak4	Intercept	-0.4557	0.173	0.44
	G	0.0255	0.074	
	isf	0.0879	<0.001	
	G:isf	-0.004	<0.05	

## Discussion

### Environmental and structural variables determining the oak regeneration performance

The results of this study showed that natural oak regeneration performance cannot be attributed to single variables, but depends on a combination of variables, which was also found by Reif and Gärtner [34]. Above all, light conditions are mentioned in literature as being important for the oak regeneration (e.g. [46–49]). Oak regeneration is also subject to high levels of browsing (e.g. [33,50]). But also competition with ground vegetation (e.g. [51,52]), and nutrient or water supply (e.g. [53]) are mentioned. All these variables were identified as being important and can be confirmed by this study (Table 2). It is the combination of the variables that creates favorable or unfavorable conditions for the occurrence of natural oak regeneration, which shows the importance of considering various variables to successfully regenerate oak naturally. These findings allow answering our first research question, namely that conditions can be identified that are favorable for natural oak regeneration.

### Differentiation among oak regeneration development stages

This study tried to differentiate ecological prerequisites for natural oak regeneration in general but especially for different development stages of the regeneration. In oak forest stands, mature trees usually begin to regenerate with an age of 30 to 40 years, with *Q*. *petraea* usually beginning slightly later than *Q*. *robur* [54,55]. During the first year, seedling growth is mainly a result of the acorn weight and size [56,57]. For this reason, oak seedlings often survive in large numbers [27] and unfavorable conditions. However, already shortly after this stage, other requirements come into play that affect the survival of the oak saplings. This shows that ontogeny has a strong impact on resource requirement as it was observed for European beech [58]. Accordingly, we found some variable combinations to be more strongly related to natural oak regeneration abundance in the different development stages than others. Their role is discussed in the following.

### Seedlings (development stage: Oak1)

For the occurrence of seedlings, the study has shown that mainly the overstory composition is of importance, since the basal area of oaks (G_OAK) and other species (G_OSP) in the overstory were important explanatory variables (Table 2). The CCA analysis showed that seedling abundance strongly decreased with increasing basal area of other species (G_OSP) and generally increased in the direction of increasing basal area of oak (G_OAK) (Fig 1). Highest regeneration abundances for Oak1 was also found where the total basal area (G) was around 25 m² ha^-1^, with high proportions of oak (G_OAK) and low proportions of other species (G_OSP). This result confirmed earlier findings for other species indicating that conspecific adult overstory density may have a positive effect on seedling density in early development stages [59]. However, in later development stages overstory tree density may have the opposite effect [59]. From other studies it is known that slightly reduced basal area, e.g. by a light shelterwood cutting could increase the survival rates of oak seedlings, due to an increased photosynthetic potential and water-use efficiency [60]. Next to the overstory composition, soil Fe content was positively associated with oak abundance (Table 2). Major et al. [61] found that red oak (*Q*. *rubra* L.) was less abundant on fertile sites with elevated soil calcium. Here, higher soil pH values and CEC values were related to higher abundances of maple, ash, and hornbeam regeneration. The findings suggest that soil conditions indirectly affect seedling abundance twofold: Firstly, competition by other tree species in the regeneration increases with increasing soil fertility. Secondly, mature oak trees, which are more likely to be cultivated on less fertile soils, predetermine oak regeneration on these sites. The reason for cultivating oaks on nutrient poor sites is because other high value timber species, for example, maple, ash, cherry and *Sorbus* species, cannot grow there. In this study, soil water content was not identified as important variable for the oak regeneration, neither when estimated as field capacity, nor following the forest site classification system. Possibly the soil water conditions were not extreme enough to see an effect. Marginal water conditions might not have been represented well, because all sites are considered suitable for oak regeneration.

### Young saplings (development stage: Oak2 and Oak3)

Sapling occurrence from development stage Oak2 and Oak3 was related to browsing (Tables 2 and 3), but low regeneration abundances could not be assigned to high browsing rates, as one would have expected (e.g. [62]). The CCA (Fig 1) showed that more oak regeneration from development stage Oak3 was found in the direction of higher browsing percentage. In any case, if many oaks occur in the regeneration also many of them are browsed. Other studies pointed out that oak seedlings and saplings are among the most attractive broadleaved tree species, especially to roe deer (*Capreolus capreolus* L.) [34,63]. It may therefore be that in some stands oak regeneration was missing due to heavy browsing, because the seedlings had already disappeared after they had been repeatedly browsed (browsing freshly germinated seedlings to death, comp. [64]). These cases may misleadingly support the conclusion that no negative impact of browsing on oak regeneration exists. For a more detailed differentiation between browsing impact and other important variables pairs of fenced and unfenced plots were established for future measurements in each of the study areas.

Studies from North America have shown that oaks are more successful where fires reduce overstory density, understory competition, and increase light penetration through the canopy, creating an environment more beneficial for the regeneration of *Q*. *rubra* [49,65–67]. Beech overstory and regeneration (BCH, bch) were important variables for Oak2 (Table 2) and increased in the direction of decreasing regeneration from Oak2. This can be interpreted as result of competition, also confirmed in North American red oak stands, where shade-tolerant species suppress oak regeneration under the absence of fire [68]. However, conifer regeneration (ocs) increased in the same direction as Oak3 (Fig 1). Soil pH was also identified as important for the abundance of Oak3 by the Boruta analysis (Table 2). Sapling abundance decreased with increasing pH (Fig 1). This finding again points to less severe competition by species such as sycamore maple (*Acer pseudoplatanus* L.) and European ash (*Fraxinus excelsior* L.) on less fertile sites, as the two competing species are strongly dependent on base saturation in early development stages [69].

This study showed that the occurrence of oak saplings from development stage Oak2 was related to light conditions (Table 2) and that abundance increased with increasing light availability (Table 3). Highest regeneration abundances for Oak2 and Oak3 were found for isf values around 20%. This supports the hypothesis that there is an optimal value of light availability for oak regeneration at this stage [47,55,70]. However, light requirement of oak increases with increasing age and size [70]. Thus, the “optimum” range of light conditions will change over time.

### Old saplings (development stage: Oak4)

Old saplings also showed a clear relation to light conditions and basal area of the stand (Table 2). The abundance of Oak4 increased with decreasing basal area of the other tree species (G_OSP) and increasing basal area of oak (G_OAK). An even stronger increase could be found with increasing isf (Fig 1). In stands of a given stand density, light transmission is higher under oak trees than under European beech [71]. The finding that increasing overstory oak density seemed to even promote oak saplings was unexpected. However, it may partly be an artefact as it may represent that high mature oak density corresponds with low density of other, more shade casting species, such as European beech. As not a single out of our 48 stands provided more than 36% isf, the positive relationship between G_OAK and abundance of oak saplings does not mean that the latter would not have benefited from light conditions beyond this value. Even though younger specimens of *Q*. *petraea* are assumed to be slightly more shade tolerant than specimens of *Q*. *robur* [72], light requirements in this study increased with age. This finding supports the assumption of increasing light demand of oak seedlings during ontogeny [70]. So far, we could not find any sign that oak seedlings and saplings are more shade tolerant under better site (i.e. soil nutrient and water supply) conditions as ecological theory suggests. However, as stated above it is very likely that our site gradient was not long enough to detect such pattern.

Addressing the second question raised in the introduction, the results of this study clearly indicate that different development stages of the oak regeneration are distinctively influenced and require different environmental conditions.

## Conclusions and Management Recommendations

The complementary statistical approaches (CCA, Boruta, and regression models) identified a few variables strongly related to the regenerations performance of oak. The analyses clearly revealed that the importance of variables changes in dependence of the development stages of oak, anticipating differences in requirements among development stages.

Competition within the regeneration layer is an important variable controlling the regeneration performance of oak. Tree, shrub, and herb species are known to interfere with oak seedlings [73–75]. Hence, replacement of oak regeneration by other species is recognized as a common problem after any kind of disturbance [51]. Since oaks are considered as light demanding species (e.g. [14]) any disturbance of the canopy cover in closed forests primarily results in improved conditions for seedlings and saplings of oak, but also for other species. Here, competing beech regeneration was of some importance. Where abundance of beech regeneration was high, oak regeneration was low and *vice versa*. Ligot et al. [30] found that beech saplings naturally outcompete oak saplings. The same trend was found for ash, even though this relationship was not identified as important explanatory variable. Hence, there seemed to be a tendency of mutual exclusion for some species indicating the importance of competition as the driving biotic variable of forest development (compare [76]).

We conclude that a successful natural regeneration of oak is facilitated by reducing the interspecific competition. This is achieved by regenerating oak (1) with a sufficient proportion of mature oaks in the overstory in the initial stage, (2) under appropriate i. e. continuously increasing light conditions which are achieved by repeatedly reducing overstory density, and (3) on sites not as favorable for other species, e.g., sites with lower pH values and soil CEC.
